# Supplementary material for: Abundance of Bifidobacterium species in the infant gut microbiota and associations with maternal-infant characteristics in Dhaka, Bangladesh
Source: mSphere. 2025 Sep 15;10(10):e00314-25. doi: 10.1128/msphere.00314-25 (PMC12570478; doi:10.1128/msphere.00314-25)
Supplement: Supplemental Figures and Tables — Figures S1-S8 and Tables S1-S8. [file msphere.00314-25-s0001.pdf]

Abundance of *Bifidobacterium* species in the infant gut microbiota and associations with maternal-infant characteristics in Dhaka, Bangladesh

Aline C. Freitas[a,b], Grace Li[a,b], Jakaria Shawon[c*^], Huma Qamar[a], Lisa G. Pell[a], Mamun Kabir[d], Ovokeraye H. Oduaran[a], Scarlett Puebla-Barragan[a], Diego G. Bassani[a,b,e], Karen M. O'Callaghan[f], Jennifer C. Onuora[a,g], Miranda G. Loutet[a], Cole Heasley[a], Cody W. E. Starke[a], Abdullah Al Mahmud[c+], Davidson H. Hamer[h,i], Eleanor Pullenayegum[b], Md Iqbal Hossain[c], Md Muniruzzaman Siddiqui[j], Mohammad Shahidul Islam[k], Philip M. Sherman[l,m], Prakesh S. Shah[m,n], S. M. Abdul Gaffar[c], Shamima Sultana[c], Shaun K. Morris[a,b,m], Tahmeed Ahmed[c], Rashidul Haque[d], Shafiqul Alam Sarker[c], Daniel E. Roth[a,b,e,g,m]#

[a]Centre for Global Child Health, Hospital for Sick Children, Toronto, Ontario, Canada

[b]Child Health Evaluative Sciences, Hospital for Sick Children, Toronto, Ontario, Canada

[c]Nutrition Research Division, International Centre for Diarrhoeal Disease Research, Dhaka, Bangladesh

[d]Infectious Diseases Division, International Centre for Diarrhoeal Disease Research, Dhaka, Bangladesh

[e]Dalla Lana School of Public Health, University of Toronto, Toronto, Ontario, Canada

[f]Department of Nutritional Sciences, King's College London, London, United Kingdom

[g]Department of Nutritional Sciences, Faculty of Medicine, University of Toronto, Toronto, Ontario, Canada

[h]Department of Global Health, Boston University School of Public Health, Boston, MA, USA

[i]Department of Medicine, Boston University Chobanian & Avedisian School of Medicine, Boston, MA, USA

[j]Mohammadpur Fertility Services and Training Centre, Dhaka, Bangladesh

[k]Child Health Research Foundation, Dhaka, Bangladesh

[l]Cell & Systems Biology, Research Institute, Hospital for Sick Children, Toronto, Ontario, Canada

[m]Department of Paediatrics, Faculty of Medicine, University of Toronto, Toronto, Ontario, Canada

[n]Department of Pediatrics, Mt. Sinai Hospital, Toronto, Ontario, Canada

[*]Present address: Department of Oncological Sciences, University of Utah, Salt Lake City, UT, USA

[^]Present address: Huntsman Cancer Institute, Salt Lake City, UT, USA

[+]Present address: Shield Pharmaceuticals Corp., Ronkonkoma, NY, USA

#Correspondence: daniel.roth@sickkids.ca

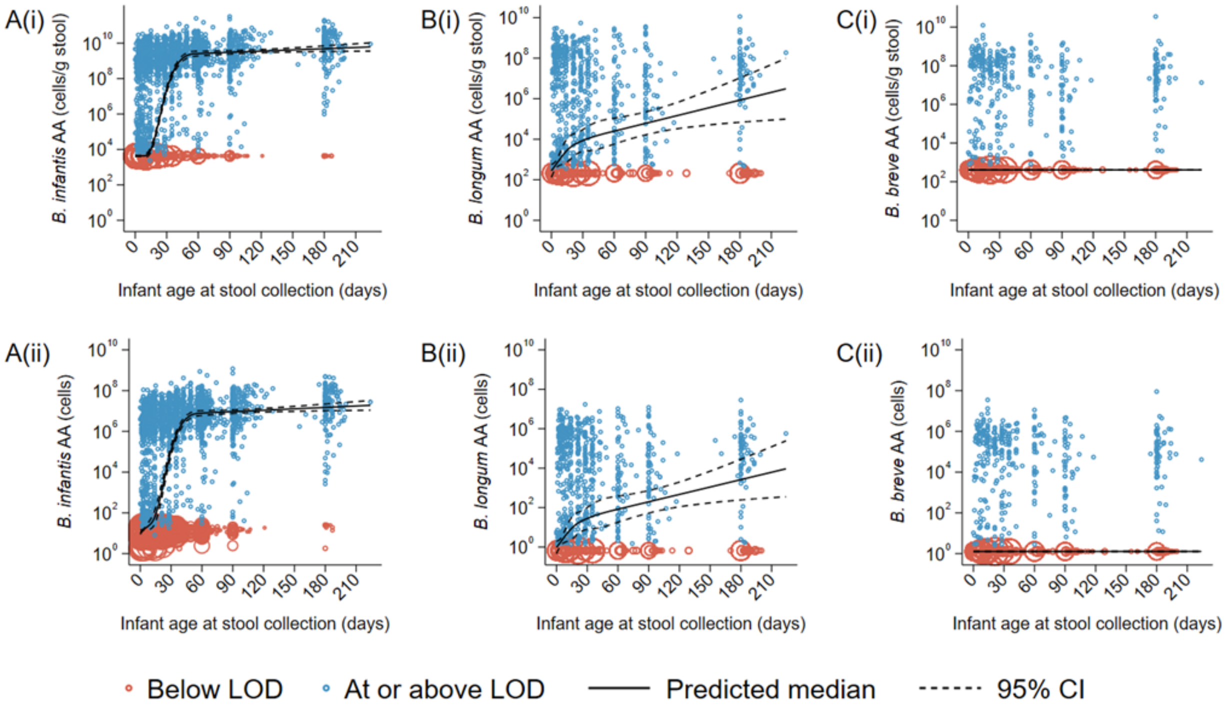

Figure S1. Sensitivity analysis: *Bifidobacterium* (sub)species trajectory in early infancy, based on different normalization approaches: per stool mass (i) or not normalized (ii).

(A) *B. infantis* (n=4613); **(B)** *B. longum* (n=1123); **(C)** *B. breve* (n=1161). Sample size (n) refers to the number of samples. The predicted median of absolute abundance (AA) and 95% confidence interval (95% CI) are based on a quantile regression model with clustered standard errors and restricted cubic splines (knots at 7, 14, 28 and 60 days of age). Samples below the assay limit of detection (LOD) were imputed as the median of one-half the LOD normalized to gram of stool, or not normalized. The size of each circle is proportional to the number of overlapping points.

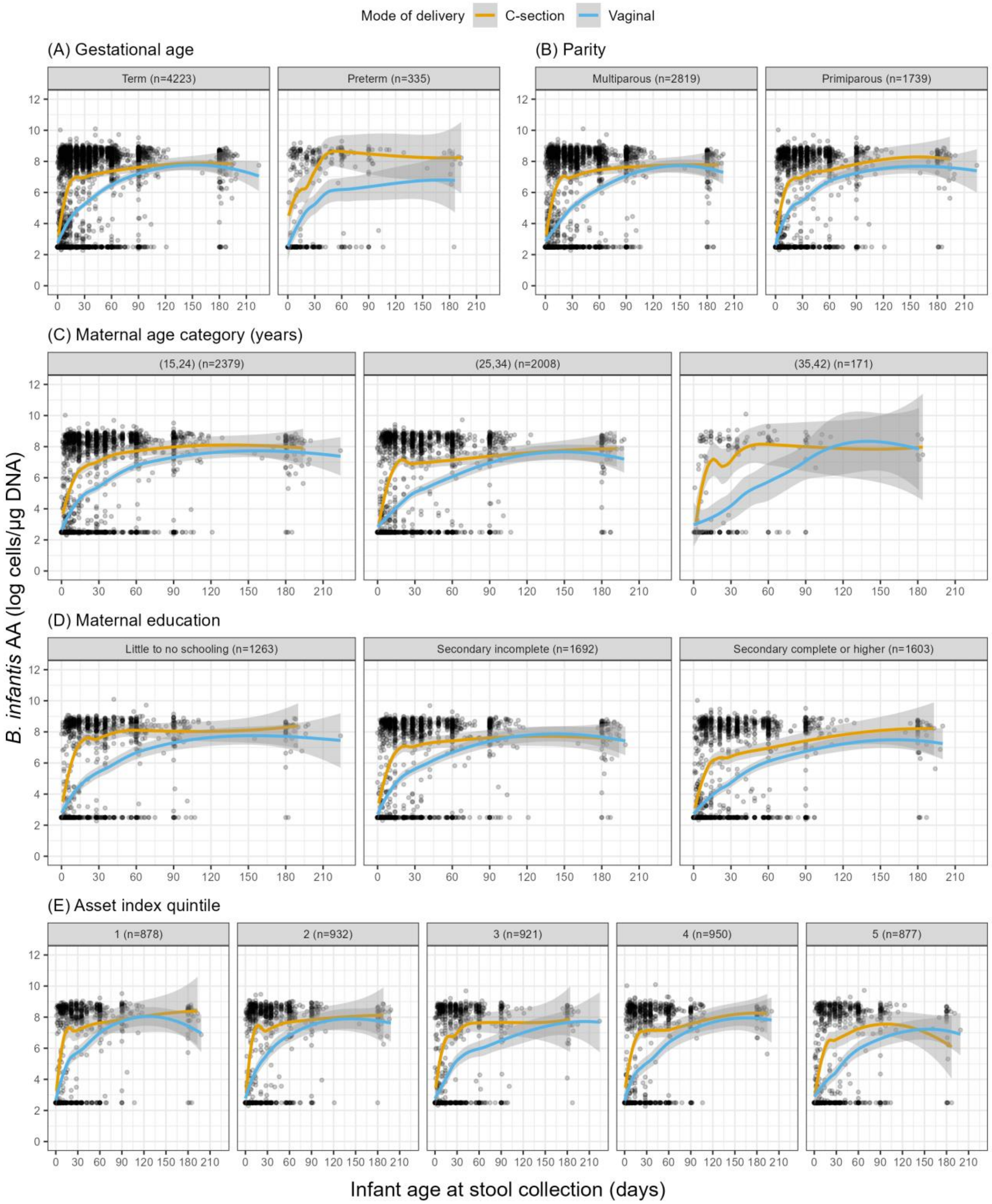

Figure S2. ***B. infantis*** **absolute abundance (AA) by mode of delivery, stratified by putative confounders.**

AA represented as loess curve (solid line) and 95% confidence interval (shade). Total samples, n=4558 (for all panels).

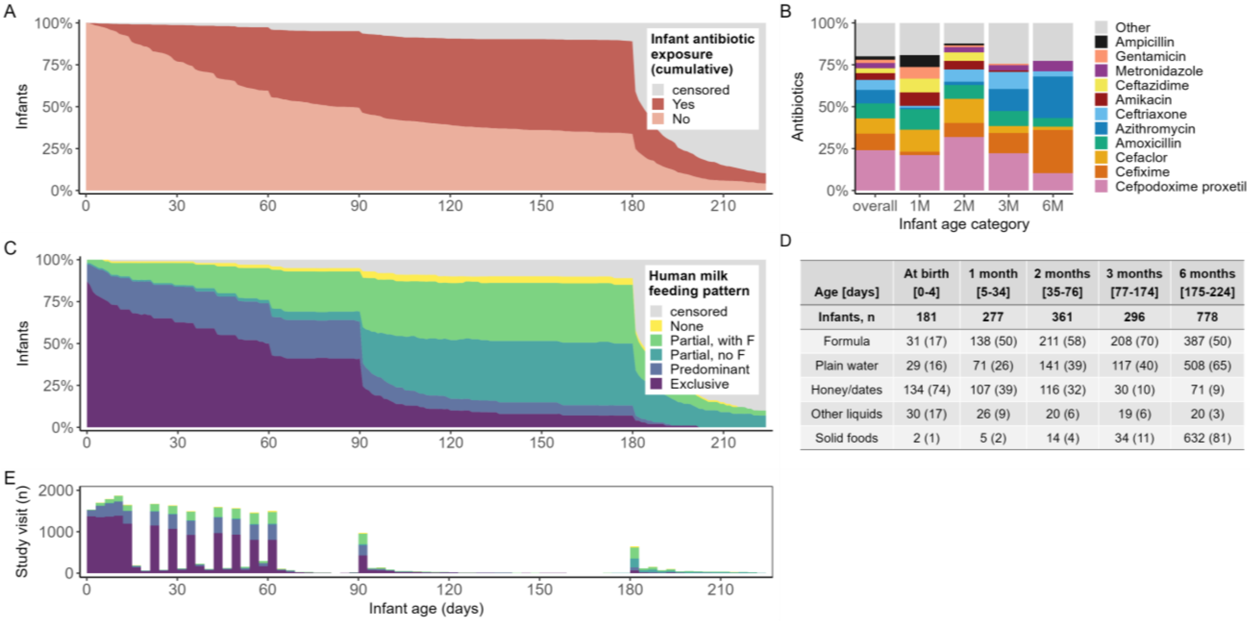

Age [days]	At birth [0-4]	1 month [5-34]	2 months [35-76]	3 months [77-174]	6 months [175-224]
**Infants, n**	**181**	**277**	**361**	**296**	**778**
Formula	31 (17)	138 (50)	211 (58)	208 (70)	387 (50)
Plain water	29 (16)	71 (26)	141 (39)	117 (40)	508 (65)
Honey/dates	134 (74)	107 (39)	116 (32)	30 (10)	71 (9)
Other liquids	30 (17)	26 (9)	20 (6)	19 (6)	20 (3)
Solid foods	2 (1)	5 (2)	14 (4)	34 (11)	632 (81)

Figure S3. Infant antibiotic exposure (A-B) and feeding pattern (C-E) up to six months of age (infants, n=1132).

(A) Cumulative antibiotic exposure ('Yes' indicates previous exposure up to that day and 'No' indicates never being exposed). **(B)** Most common systemic antibiotics reported around 1M (0-34 days), 2M (35-76 days), 3M (77-174 days), and 6M (175-224 days) of age (M=month). Counts for antibiotics were determined at each calendar week (0-6, 7-13, 14-20, etc.), thus antibiotics reported in multiple visits within a week were counted only once (for example, if amoxicillin was reported at two scheduled visits on days 1 and 6, amoxicillin would be counted once at the first week of age); then counts were binned by age windows and the proportions calculated for plotting. The top 5 antibiotics in any age window were reported in addition to gentamicin and ampicillin (which are relevant for this population based on the WHO recommendation for management of infections in infants (0–59 days old) when referral to hospital is possible (1). **(C)** Cumulative human milk feeding pattern from birth up until the next scheduled study visit. The artificial plateau in the feeding pattern along some intervals (e.g., 60-90 and 90-180 days) reflects the fewer study visits within those periods since most visits after two months of age happened around 60, 90 and 180 days of age. F=infant formula. **(D)** Most common foods reported among infants who did not consume human milk exclusively (values in the table represent the number and percentage of infants who consumed each food category, n (%)). **(E)** Number of study visits for which feeding patterns were ascertained (bin width = 3 days). The 'censored' group in panels A/C corresponds to the proportion of infants from which there were no data collected due to loss to follow up (that is, infants did not complete all expected visits) or study exit (scheduled at day 180 but allowed to happen afterwards).

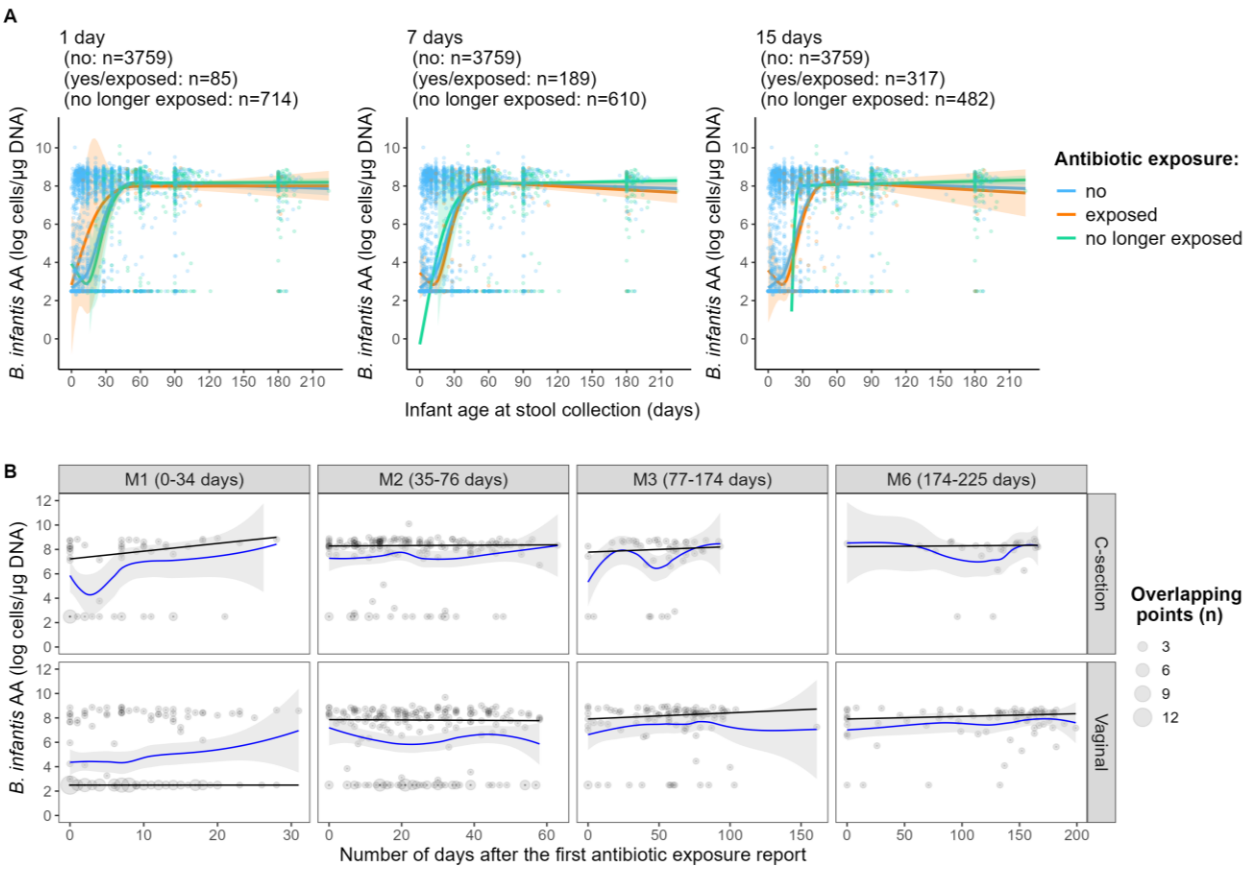

Figure S4. Sensitivity analysis: short-term association of antibiotic exposure with *B. infantis* absolute abundance (AA).

(A) *B. infantis* AA trajectory by antibiotic exposure, where exposure status was defined as: 'yes/exposed' (stool samples collected up to 1, 7 or 15 days after each infant's first antibiotic exposure), 'no longer exposed' (samples collected more than 1, 7 or 15 days after the first antibiotic exposure), 'no' (samples from participants who were never exposed to antibiotics). Sample sizes (n) are depicted at the top of each plot. The predicted median of AA (solid line) and 95% confidence interval (95% CI, shade) are based on a quantile regression model with clustered standard errors and restricted cubic splines (knots at 7, 14, 28 and 60 days of age), adjusted for confounders (see Methods). **(B)** *B. infantis* AA across stool samples collected at different days post each infant's first antibiotic exposure, stratified by mode of delivery and age window (i.e., infant age at sample collection; M=month). Each circle represents a sample collected within an infant age stratum and at a certain number of days after the first antibiotic exposure (depicted at the x-axis). The size of each circle is proportional to the number of overlapping points. Fitted lines denote median (quantile regression, solid black line) and loess curve (solid blue line). Sample size: C-section: M1=55, M2=152, M3=48, M6=29; Vaginal delivery: M1=140, M2=194, M3=102, M6=79.

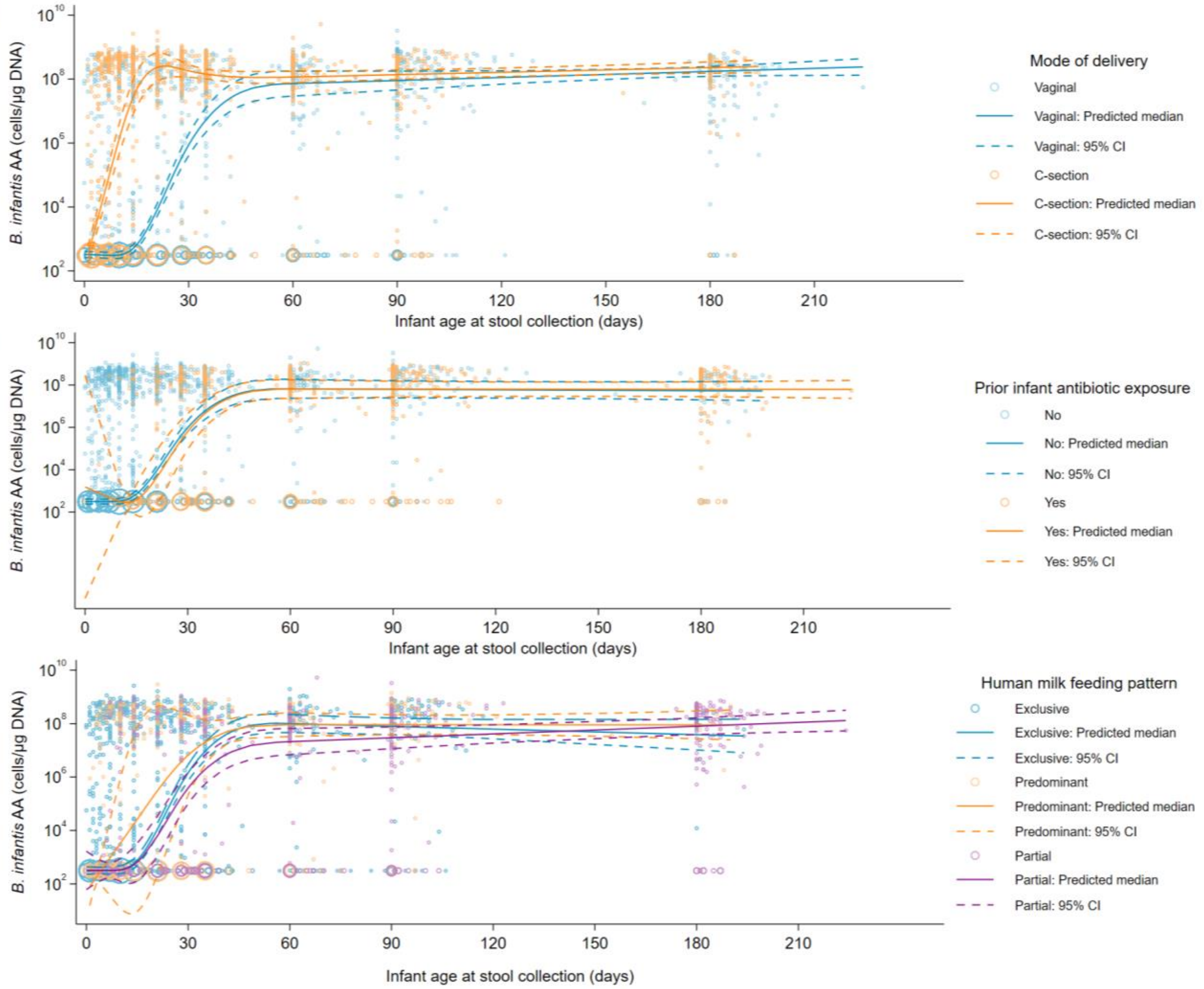

Figure S5. Sensitivity analysis: *B. infantis* absolute abundance trajectory by infant-maternal characteristics, restricted to participants from schedules A and B.

(A) Mode of delivery (n=3271); **(B)** Infant antibiotic exposure (n=3271); **(C)** Infant feeding pattern (n=3238). Sample size (n) refers to the number of samples. The predicted median of absolute abundance (AA) and 95% confidence interval (95% CI) are based on a quantile regression model with clustered standard errors and restricted cubic splines (knots at 7, 14, 28 and 60 days of age), adjusted for confounders. Samples below the assay limit of detection (LOD) were imputed as the median of one-half the LOD normalized to µg DNA. The size of each circle is proportional to the number of overlapping points. The 'none' category for infant feeding pattern was dropped due to small sample size (n=20 of 3238).

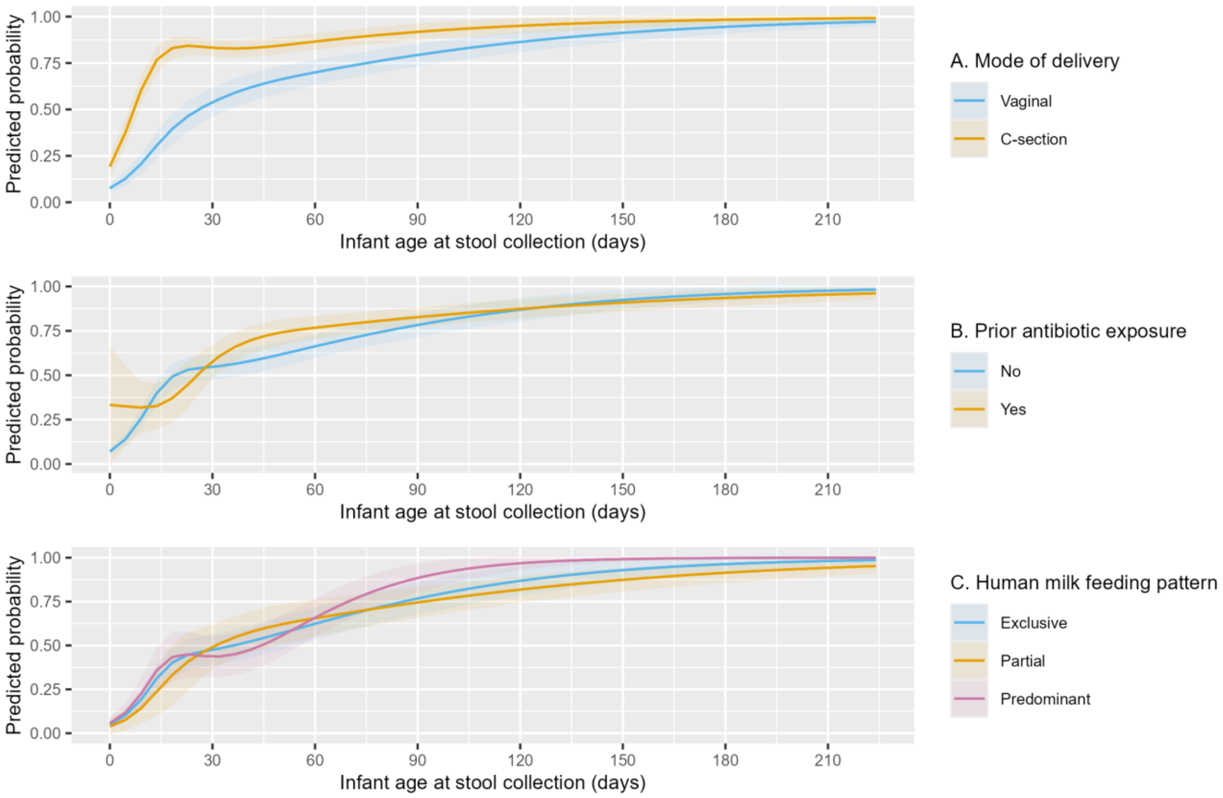

Figure S6. Probability of high absolute abundance of *B. infantis*, by infant-maternal characteristics.

(A) Mode of delivery (n=4558); **(B)** Infant antibiotic exposure (n=4558); **(C)** Infant feeding pattern (n=4485). Sample size (n) refers to samples. The predicted probability of high *B. infantis* AA (≥6 log cells/µg DNA) (solid line) and 95% confidence interval (shade) are based on a Generalized Estimating Equations (GEE) model with restricted cubic splines (knots at 7, 14, 28 and 60 days of age), adjusted for confounders. The 'none' category for feeding pattern was dropped due to small sample size (n=36 of 4521).

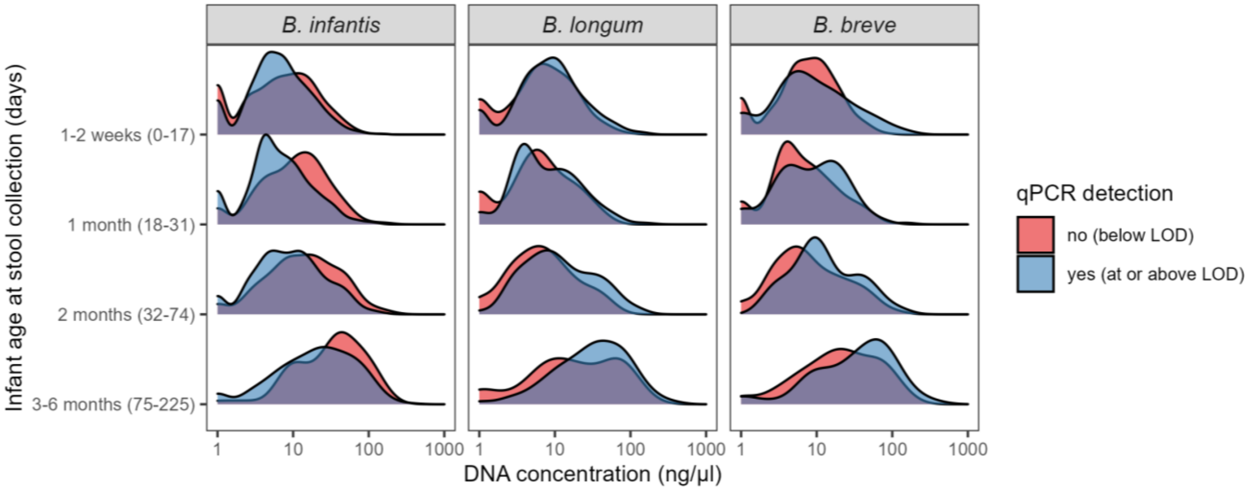

Figure S7. DNA concentration distribution and qPCR detection status of *B. infantis, B. longum* and *B. breve*.

Only samples tested for all three targets were included in this analysis (n=1120). The scale is fixed across all panels. LOD: limit of detection of the qPCR assay.

A

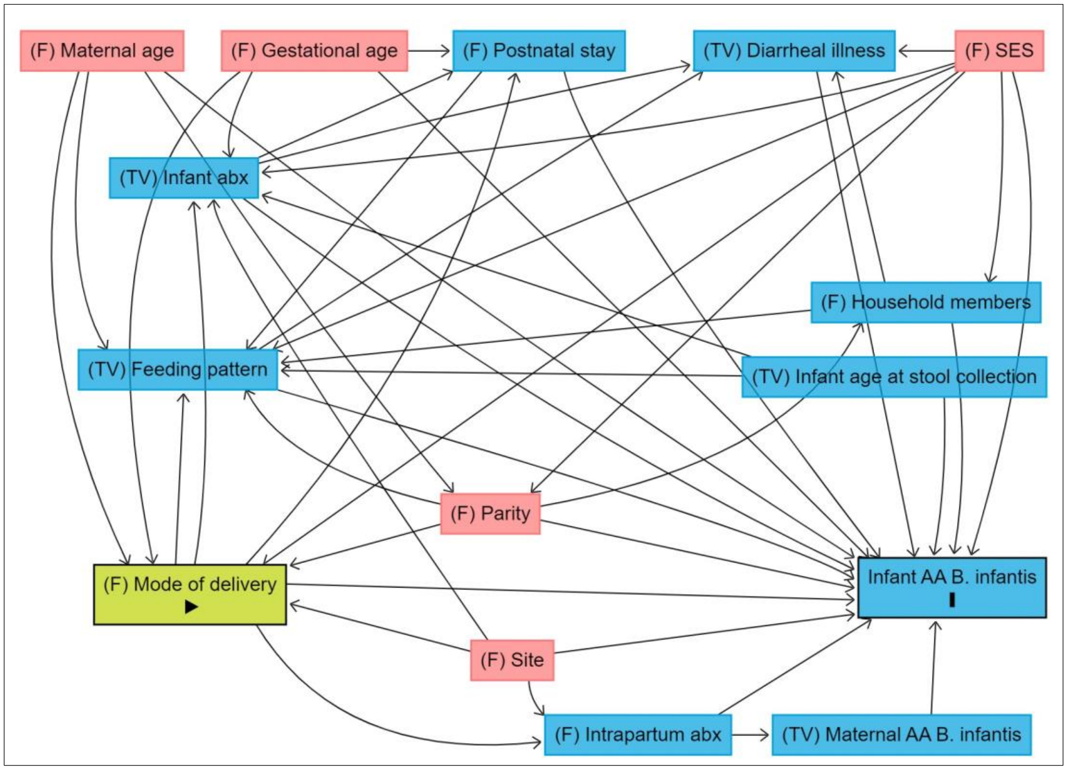

B

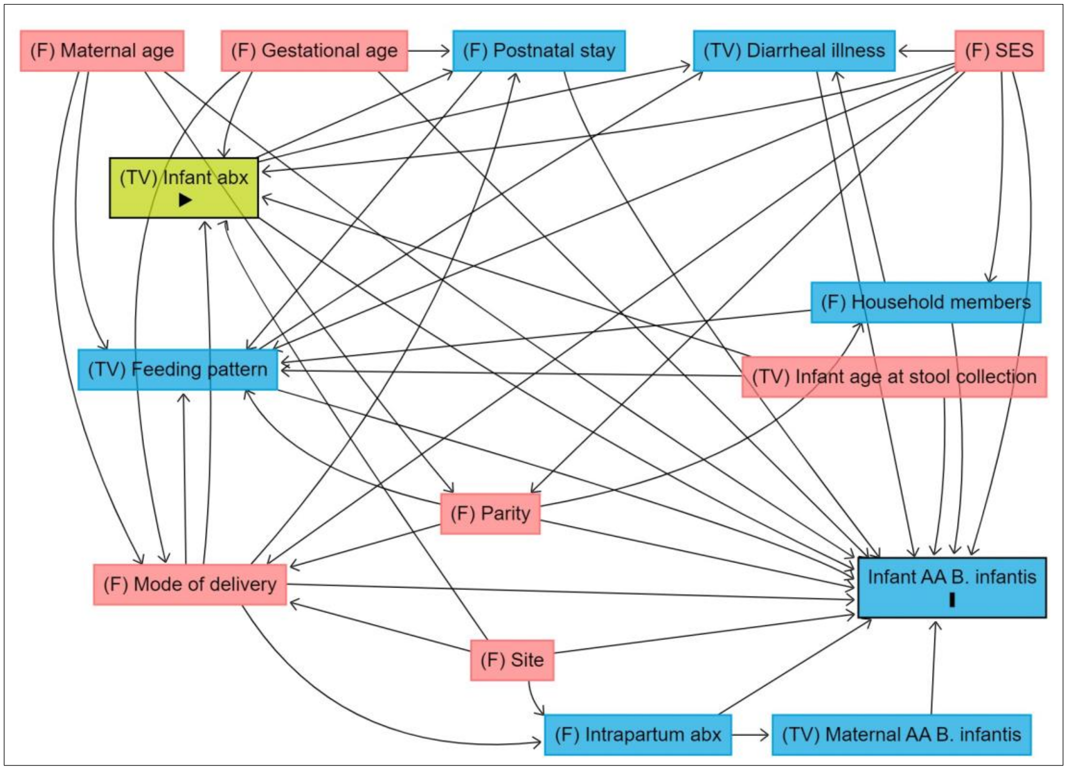

(Figure S8. continued on next page)

C

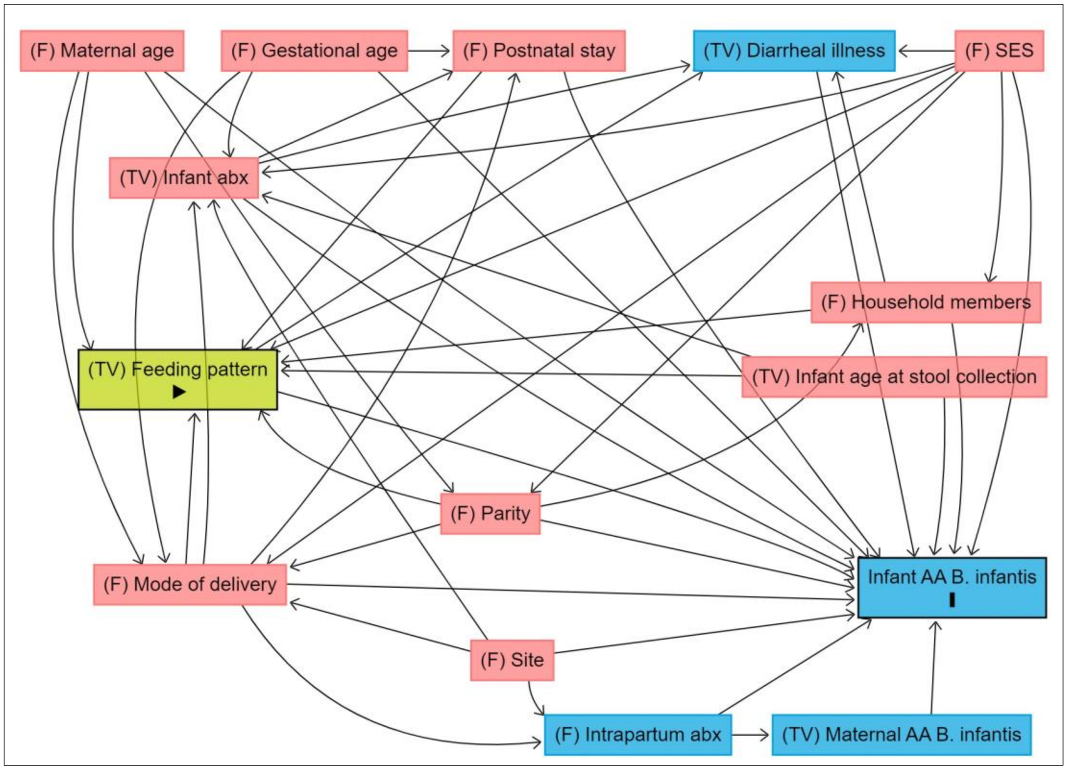

Legend:

exposure
outcome
ancestor of exposure
ancestor of outcome
ancestor of exposure *and* outcome

Figure S8. Directed Acyclic Graphs (DAG) of the association between *B. infantis* absolute abundance (AA) in infant stool samples and exposures.

(A) Mode of delivery; **(B)** Infant antibiotic exposure; **(C)** Infant feeding pattern. TV: time varying variable; F: fixed variable; SES: socioeconomic status (represented by maternal education and asset index); abx: antibiotics.

Table S1. Infant, maternal, and household characteristics, by enrollment study site.

	MCHTI	MFSTC	*p-value* [k]
Delivery and infant characteristics: infants, n (%) [a]	**452 (40)**	**680 (60)**	
Infant age at enrollment (days), median (25th, 75th) [min, max]	1 (1, 2) [0, 4]	1 (0, 1) [0, 4]	<0.001
Gestational age at delivery (weeks), median (25th, 75th)	39.3 (38.3, 40.1)	39.1 (38.1, 40.1)	0.5
Term (≥ 37 weeks), n (%)	429 (95)	612 (91)	0.01
Preterm (< 37 weeks), n (%)	23 (5)	62 (9)	
Mode of delivery, n (%)			<0.001
Vaginal	171 (38)	351 (52)	
C-section	281 (62)	329 (48)	
Birth weight [b] (g), median (25th, 75th)	2840 (2580, 3060)	2860 (2600, 3150)	0.2
Sex: male infants, n (%)	208 (46)	331 (49)	0.4
Singletons, n (%)	444 (98)	671 (99)	0.7
Postnatal hospital stay (days), median (25th, 75th)	4 (1, 5)	2 (1, 4)	<0.001
Vaginal delivery	1 (1, 1)	1 (0, 1)	<0.001
C-section delivery	5 (4, 5)	4 (4, 4)	<0.001
Infant antibiotic exposure [c], n (%)			
Up to 1 month of age	125 (28)	100 (15)	<0.001
Up to 2 months of age	233 (53)	222 (33)	<0.001
Up to 6 months of age	329 (77)	300 (51)	<0.001
Human milk feeding pattern [d], n (%)	Exclusive | Predominant Partial | None	Exclusive | Predominant Partial | None	
At or near enrollment	386 (86) | 55 (12) 10 (2.2) | 0 (0)	602 (89) | 56 (8.3) 16 (2.4) | 2 (0.3)	0.1
At 1 month of age	241 (59) | 99 (24) 67 (16) | 4 (1.0)	437 (69) | 118 (19) 69 (11) | 7 (1.1)	0.004
At 2 months of age	190 (43) | 116 (26) 123 (28) | 11 (2.5)	386 (58) | 150 (23) 117 (18) | 11 (1.7)	<0.001
At 6 months of age	25 (6.1) | 45 (11) 314 (76) | 28 (6.8)	54 (11) | 25 (5.1) 400 (82) | 9 (1.8)	<0.001
Diarrheal illness [e], n (%)			
Up to 1 month of age	31 (7.0)	12 (1.8)	<0.001
Up to 2 months of age	59 (13)	29 (4.3)	<0.001
Up to 6 months of age	126 (29)	69 (12)	<0.001
Mother and household characteristics, n (%)	**448 (40)**	**677 (60)**	
Maternal age (years), median (25th, 75th)	24 (20, 27)	24 (20, 28)	0.7
Parity, median (25th, 75th)	2 (1, 2)	2 (1, 2)	>0.9
Primiparous, n (%)	185 (41)	269 (40)	0.6
Maternal peripartum antibiotics [f], n (%)			<0.001
None	24 (5.4)	33 (4.9)	
Intrapartum only	16 (3.6)	49 (7.2)	
Postpartum only	256 (57)	548 (81)	
Both intrapartum and postpartum	152 (34)	47 (6.9)	
Maternal education, n (%)			<0.001
Little to no schooling [g]	95 (21)	223 (33)	
Secondary incomplete	173 (39)	206 (30)	
Secondary complete or higher	180 (40)	248 (37)	
Asset index quintile [h], n (%)			0.002
1 (lowest wealth)	64 (14)	142 (21)	
2	90 (20)	134 (20)	
3	82 (18)	145 (22)	
4	119 (27)	124 (18)	
5 (highest wealth)	89 (20)	126 (19)	
Household size [i], median (25th, 75th)	5 (4, 7)	5 (4, 6)	<0.001
One or more household members ≤ 5 years old [j], n (%)	157 (37)	196 (31)	0.05

[a] Missing data: gestational age (n=6, 0.5%); infant antibiotic exposure (n=13 (1.1%), n=22 (1.9%), n=117 (10%) for 1, 2 and 6 months of age, respectively); human milk feeding pattern (n=5 (0.4%), n=90 (8%), n=28 (2.5%), n=232 (20%), for 1, 2 and 6 months of age, respectively); diarrheal illness (n=13 (1.1%), n=23 (2%), n=116 (10%) for 1, 2 and 6 months of age, respectively); asset index (n=10, 0.9%); household size (n=17, 1.5%); members ≤ 5 years old (n=58, 5.2%).

[b] Weight measured by study personnel was used as a birthweight proxy for four infants where there was >15% difference between birthweight and weight measured within 1-4 days of birth; for three infants, weight was measured within 1 day of birth and, for one infant, weight measured within 4 days of birth was used.

[c] Antibiotic exposure status was derived as any exposure versus no exposure within age windows around the scheduled visits at 1 month (25-31 days), 2 months (46-74 days) and 6 months (173-225 days). Data were considered missing for an infant if their last study visit happened before the age window cut-off.

[d] Human milk feeding pattern was derived based on the infant's feeding status at the closest scheduled visit available at enrollment (0-4 days), and at 1, 2 and 6 months (same age windows as defined in 'c'). Data were considered missing for an infant if there were no study visits within the age window.

[e] Diarrheal illness status was defined as any previous episode of diarrhea (versus never), reported at scheduled visits around 1, 2 and 6 months of age (same age windows and criteria for missing data as defined in 'c').

[f] Intrapartum refers to any antibiotics administered before labour (at hospital admission in which delivery occurred), during labour and/or in the operating theatre, but prior to delivery. Postpartum refers to antibiotics administered after the mother has delivered the infant.

[g] Includes women with no formal education, and incomplete and completed primary school.

[h] Asset index scores were generated using Principal Components Analysis (PCA) for all participants enrolled in the observational cohort study (n=1886) and in a concurrently running trial (n=519) at the same study sites with the same eligibility criteria. The score is a summary measure of household wealth based on ownership of the following assets: electricity, radio, television, almirah, fan, table, chair, fridge, pump, freezer, phone, animals, mobile, watch, computer, autobike, bicycle, rickshaw and a vehicle (2).

[i] Number of people that sleep in the same house as the infant enrolled in the study, including the infant's twin, if applicable, but excluding the infant enrolled in the study.

[j] Excludes the infant enrolled in the study.

[k] P-values: Wilcoxon rank-sum tests were used to compare medians of continuous variables, which were all non-normally distributed; Chi-square tests were used to compare categorical variables when expected counts were ≥ 5. Fisher's exact tests were used to compare categorical variables when expected counts were < 5.

Table S2. Sample size for qPCR analysis, by enrollment schedule (A, B, C).

		B. infantis				***B. longum***				***B. breve***			
**Scheduled visit (days since birth)**	**Infant age in days (min-max)**	**Total**	**A**	**B**	**C**	**Total**	**A**	**B**	**C**	**Total**	**A**	**B**	**C**
0-1	0-2	421	51	226	144	63	4	58	1	69	4	58	7
3-4	3-5	486	55	231	200	80	12	67	1	94	12	67	15
6-7	6-8	476	71	273	132	110	22	88	0	122	22	88	12
10	9-10	394	66	216	112	73	19	54	0	77	19	54	4
14	11-17	453	76	304	73	113	19	94	0	114	19	94	1
21	18-24	398	68	254	76	119	28	91	0	118	28	90	0
28	25-31	413	65	250	98	116	26	90	0	119	26	93	0
35	32-45	486	70	261	155	115	22	93	0	113	22	91	0
60	46-74	597	58	245	294	100	20	80	0	101	20	81	0
90	75-130	324	64	244	16	108	26	82	0	108	26	82	0
180	131-225	165	41	124	0	126	34	92	0	126	34	92	0
**Total infant samples**	**-**	**4613**	685	2628	1300	**1123**	232	889	2	**1161**	232	890	39
**Total infants**	**-**	**1132**	75	287	770	**364**	75	287	2	**399**	75	287	37
**Total mother [a]**	0-7	**491**	66	164	261	-	-	-	-	-	-	-	-

[a] Sample size refers to 491 mothers with 495 infants (i.e., 4 twin pairs), where each mother provided one sample within the specified age window.

Table S3. Absolute abundance of *B. infantis*, *B. longum* and *B. breve* at discrete infant ages. [a]

Target	Age (days)	Predicted median: Log cells per µg DNA (95% CI)	Predicted median: Log cells per gram stool (95% CI)	Predicted median: Log cells (95% CI)
B. infantis	0	2.49 (2.45, 2.54)	3.63 (3.58, 3.69)	1.06 (0.96, 1.16)
	7	2.49 (2.45, 2.54)	3.63 (3.58, 3.69)	1.32 (1.18, 1.46)
	14	2.76 (2.64, 2.87)	3.89 (3.75, 4.02)	1.78 (1.45, 2.12)
	21	4.14 (4.01, 4.26)	5.21 (5.05, 5.38)	3.08 (2.74, 3.43)
	28	5.94 (5.83, 6.06)	6.96 (6.79, 7.12)	4.67 (4.42, 4.92)
	35	7.25 (7.14, 7.35)	8.22 (8.06, 8.38)	5.81 (5.63, 6.00)
	42	7.98 (7.88, 8.08)	8.95 (8.79, 9.11)	6.47 (6.32, 6.63)
	60	8.38 (8.30, 8.47)	9.41 (9.28, 9.55)	6.90 (6.78, 7.02)
	90	8.30 (8.23, 8.37)	9.48 (9.38, 9.58)	6.97 (6.88, 7.06)
	180	8.06 (7.93, 8.19)	9.69 (9.53, 9.85)	7.18 (7.01, 7.34)
B. longum	0	1.24 (0.90, 1.58)	2.44 (2.13, 2.76)	-0.03 (-0.34, 0.29)
	7	1.82 (1.56, 2.09)	2.94 (2.69, 3.19)	0.46 (0.21, 0.70)
	14	2.36 (1.78, 2.95)	3.40 (2.85, 3.96)	0.91 (0.37, 1.45)
	21	2.70 (2.12, 3.29)	3.71 (3.15, 4.26)	1.21 (0.66, 1.76)
	28	2.89 (2.37, 3.42)	3.89 (3.37, 4.41)	1.40 (0.87, 1.93)
	35	3.02 (2.43, 3.61)	4.03 (3.43, 4.64)	1.55 (0.92, 2.18)
	42	3.11 (2.47, 3.75)	4.15 (3.48, 4.82)	1.67 (0.98, 2.37)
	60	3.25 (2.65, 3.86)	4.40 (3.76, 5.04)	1.92 (1.26, 2.58)
	90	3.44 (2.83, 4.05)	4.78 (4.22, 5.35)	2.29 (1.72, 2.86)
	180	4.01 (2.53, 5.48)	5.93 (4.84, 7.02)	3.42 (2.39, 4.45)
B. breve [b]	0	1.51	2.61	0.11
	7	1.51	2.61	0.11
	14	1.51	2.61	0.11
	21	1.51	2.61	0.11
	28	1.51	2.61	0.11
	35	1.51	2.61	0.11
	42	1.51	2.61	0.11
	60	1.51	2.61	0.11
	90	1.51	2.61	0.11
	180	1.51	2.61	0.11

[a] Predicted median of absolute abundance (AA) and 95% confidence interval (95% CI) are based on a quantile regression model with clustered standard errors and restricted cubic splines (knots at 7, 14, 28 and 60 days of age). AA was treated as the dependent continuous variable, and infant age was the primary independent continuous variable. Samples with values below the assay limit of detection (LOD) were imputed as a single value represented by the median of half the LOD (from samples for which LOD imputation was applied) normalized to µg DNA, gram of stool or not normalized. Number of samples: *B. infantis* (n=4613); *B. longum* (n=1123); *B. breve* (n=1161).

[b] The predicted median AA in all ages was equivalent to the imputed value since the majority of data points were below LOD, therefore, given the little variation in AA, measures of precision could not be estimated.

Table S4. Absolute abundance of *B. infantis* at discrete infant ages, by mode of delivery, infant antibiotic exposure and infant feeding pattern. [a]

**Mode of delivery (samples, n=4558)**			
	**Predicted median: log cells per µg DNA (95% CI)**		
**Age (days)**	**Vaginal (reference)**	**C-section**	**Difference between groups**
0	2.51 (2.47, 2.55)	1.64 (1.54, 1.74)	-0.86 (-0.97, -0.75)
7	2.49 (2.46, 2.52)	4.69 (4.50, 4.87)	2.20 (2.02, 2.39)
14	2.70 (2.63, 2.78)	7.37 (7.08, 7.66)	4.67 (4.37, 4.97)
21	3.95 (3.80, 4.09)	8.44 (8.19, 8.69)	4.50 (4.21, 4.78)
28	5.58 (5.36, 5.81)	8.46 (8.33, 8.59)	2.88 (2.62, 3.14)
35	6.78 (6.49, 7.06)	8.35 (8.27, 8.43)	1.58 (1.29, 1.87)
42	7.46 (7.16, 7.77)	8.29 (8.20, 8.38)	0.83 (0.51, 1.15)
60	7.91 (7.62, 8.20)	8.27 (8.18, 8.36)	0.36 (0.06, 0.66)
90	7.99 (7.78, 8.20)	8.29 (8.22, 8.36)	0.30 (0.08, 0.53)
180	8.23 (8.10, 8.35)	8.35 (8.20, 8.51)	0.13 (-0.07, 0.33)

**Infant antibiotic exposure (samples, n=4558)**			
	**Predicted median: log cells per µg DNA (95% CI)**		
**Age (days)**	**No exposure (reference)**	**Prior exposure**	**Difference between groups**
0	2.67 (2.46, 2.87)	3.08 (1.65, 4.52)	0.50 (-0.93, 1.93)
7	3.01 (2.74, 3.28)	2.86 (2.35, 3.37)	-0.06 (-0.58, 0.45)
14	3.52 (3.03, 4.02)	2.91 (2.39, 3.43)	-0.53 (-1.15, 0.10)
21	4.76 (4.30, 5.22)	4.11 (3.45, 4.77)	-0.57 (-1.32, 0.18)
28	6.23 (5.93, 6.53)	5.78 (5.32, 6.24)	-0.37 (-0.88, 0.14)
35	7.27 (7.05, 7.50)	6.99 (6.69, 7.30)	-0.20 (-0.52, 0.13)
42	7.86 (7.65, 8.08)	7.69 (7.44, 7.93)	-0.09 (-0.33, 0.14)
60	8.19 (7.98, 8.40)	8.10 (7.88, 8.32)	-0.01 (-0.19, 0.18)
90	8.14 (7.96, 8.31)	8.10 (7.92, 8.27)	0.04 (-0.11, 0.20)
180	7.97 (7.74, 8.21)	8.10 (7.89, 8.30)	0.20 (-0.11, 0.52)

**Infant feeding pattern (samples, n=4485)**					
	**Predicted median: log cells per µg DNA (95% CI)**				
**Age (days)**	**Exclusive human milk (reference)**	**Predominant human milk**	**Partial human milk**	**Difference between Predominant & Exclusive**	**Difference between Partial & Exclusive**
0	2.68 (2.52, 2.83)	1.82 (1.06, 2.57)	2.39 (1.03, 3.75)	-0.92 (-1.66, -0.18)	-0.29 (-1.63, 1.05)
7	2.89 (2.71, 3.07)	3.77 (2.48, 5.06)	3.00 (2.33, 3.68)	0.82 (-0.50, 2.14)	0.11 (-0.57, 0.79)
14	3.31 (3.01, 3.61)	5.58 (2.71, 8.45)	3.72 (1.51, 5.93)	2.21 (-0.69, 5.11)	0.41 (-1.82, 2.64)
21	4.59 (4.31, 4.88)	6.75 (4.07, 9.43)	4.89 (2.68, 7.10)	2.09 (-0.61, 4.79)	0.29 (-1.94, 2.51)
28	6.18 (5.96, 6.40)	7.39 (5.86, 8.93)	6.14 (4.80, 7.47)	1.15 (-0.40, 2.70)	-0.04 (-1.39, 1.30)
35	7.31 (7.11, 7.51)	7.79 (7.14, 8.44)	7.02 (6.38, 7.66)	0.42 (-0.24, 1.08)	-0.29 (-0.94, 0.37)
42	7.94 (7.74, 8.14)	8.02 (7.78, 8.26)	7.53 (7.24, 7.83)	0.02 (-0.24, 0.28)	-0.41 (-0.73, -0.09)
60	8.27 (8.08, 8.45)	8.20 (7.97, 8.44)	7.87 (7.65, 8.08)	-0.13 (-0.38, 0.12)	-0.40 (-0.62, -0.18)
90	8.15 (8.00, 8.31)	8.30 (8.12, 8.47)	7.93 (7.76, 8.11)	0.08 (-0.12, 0.28)	-0.22 (-0.41, -0.03)
180	7.81 (7.47, 8.16)	8.57 (7.99, 9.15)	8.13 (7.93, 8.32)	0.69 (0.02, 1.37)	0.31 (-0.08, 0.70)

[a] Predicted median of absolute abundance (AA) and 95% confidence interval (95% CI) are based on a quantile regression model with clustered standard errors and restricted cubic splines (knots at 7, 14, 28 and 60 days of age), adjusted for confounders [b]. AA was treated as the dependent continuous variable and exposure was included as a main effect and in an interaction term with chronologic age of the infants. Samples with values below the assay limit of detection (LOD) were imputed as a single value represented by the median of half the LOD normalized to µg DNA. The 'none' category for feeding pattern was dropped due to small sample size (n=36 of 4521).

[b] Each model was adjusted for the following covariates: *Mode of delivery*: Study site (binary), Gestational age at birth (continuous), Maternal age (continuous), Parity (binary), Maternal education (categorical), Asset index (continuous); all variables are time-fixed. *Infant antibiotic exposure*: Study site (binary), Gestational age at birth (continuous), Mode of delivery (binary), Maternal education (categorical), Asset index (continuous); all variables are time-fixed. *Infant feeding pattern*: Study site (binary), Gestational age at birth (continuous), Mode of delivery (binary), Postnatal hospital stay (continuous), Maternal age (continuous), Parity (binary), Maternal education (categorical), Asset index (continuous), Household size (continuous); all variables are time-fixed.

Table S5. Candidate mediators (M) of the association between mode of delivery (MOD) and *B. infantis* absolute abundance (AA). [a]

Candidate mediator	Age (days)	Samples (n)	Infants (n)	Model description and Coefficient (95% CI) [b]			
				AA ~ MOD	AA ~ MOD + M	AA ~ M	M ~ MOD
				Coefficient of MOD	Coefficient of MOD	Coefficient of M	Coefficient of MOD [c]
Length of postnatal hospital stay (days) [d]	3-17	1700	743	5.38 (4.84, 5.93)	5.38 (4.85, 5.92)	0.86 (0.47, 1.25)	4.66 (4.15, 5.22)
	3-31	2495	851	5.61 (5.42, 5.80)	5.61 (5.38, 5.84)	1.18 (1.03, 1.34)	4.74 (4.25, 5.29)
	3-45	2997	939	5.65 (5.52, 5.78)	5.65 (5.46, 5.84)	1.25 (1.14, 1.36)	4.82 (4.33, 5.36)
	3-17	743 [e]	743	4.89 (3.89, 5.89)	4.89 (3.46, 6.32)	0.79 (0.47, 1.11)	4.66 (4.15, 5.22)
	3-31	851 [e]	851	5.51 (5.22, 5.80)	5.51 (5.10, 5.92)	1.01 (0.76, 1.25)	4.74 (4.25, 5.29)
Maternal intrapartum antibiotics (yes)	3-17	1809	792	4.71 (3.72, 5.69)	4.71 (3.72, 5.69)	0.64 (-0.79, 2.07)	11.55 (6.99, 19.09)
	3-31	2604	893	5.40 (5.08, 5.72)	5.40 (5.08, 5.73)	2.22 (-2.14, 6.58)	11.59 (7.19, 18.67)
	3-45	3106	973	5.54 (5.35, 5.74)	5.54 (5.35, 5.74)	3.91 (1.30, 6.53)	10.95 (6.94, 17.27)

[a] All models were adjusted for study site and were restricted to samples collected at age windows within approximately the first month of age (samples collected at days 0-2 were dropped since they had low AA variability due to overall low detection rate). An interaction term with age (restricted cubic splines) was not added to facilitate interpretation of the coefficients. In all models involving MOD, the reference group was 'Vaginal delivery'. All models with *B. infantis* AA as outcome were based on a quantile regression model. Rows within the same 'candidate mediator' panel represent models restricted to different age windows.

[b] Model description: outcome ~ exposure, where the + sign indicates addition of candidate mediator into the model (if applicable). Example: AA ~ MOD + M (6th column in the table) refers to model assessing the effect of mode of delivery on *B. infantis* AA, adjusted for the candidate mediator (as listed in the first column).

[c] Models with postnatal hospital stay as outcome were based on a Poisson regression, and the coefficients are shown as incidence rate ratio. Models with intrapartum antibiotic as outcome were based on a logistic regression, and the coefficients are shown as odds ratio.

[d] Analysis excluded samples collected prior to hospital discharge.

[e] Included only the first post-discharge sample.

Table S6. Relative abundance (RA) (%) of bifidobacteria species, overall and by infant age. [a]

Species	M1 (0-34 days)	M2 (35-76 days)	M3 (77-174 days)	M6 (174-193 days)	Overall mean RA	Maximum RA
B. longum subsp*. infantis*	32.3	54.6	60.1	66.2	41.5	99.2
B. longum subsp*. longum*	7.3	8.2	4.7	2.6	6.9	77.0
B. longum unclassified	1.2	2.3	1.9	0	1.4	31.5
B. breve	9.0	10.2	5.2	5.5	8.6	98.5
B. catenulatum	4.0	2.5	1.1	0.3	3.2	96.0
B. bifidum	0.4	2.9	1.3	6.9	1.4	90.8
B. dentium	1.0	1.7	0.4	0	1.0	88.0
B. pseudocatenulatum	0.2	0.2	0	0	0.2	28.4
B. adolescentis	0.02	0.05	0.6	0	0.09	21.1
B. ruminantium	0.006	0	0	0	0.004	1.2
B. angulatum	0	0	0	0	0	0.10
B. psychraerophilum	0	0.001	0	0	0	0.07
B. merycicum	0	0	0.002	0	0	0.06
B. animalis	0	0	0	0	0	0
B. crudilactis	0	0	0	0	0	0
B. pseudolongum	0	0	0	0	0	0
B. pullorum	0	0	0	0	0	0

[a] RA was based on metagenomic analysis using MetaPhlAn4, restricted to samples from schedule A (n=55 infants, n=332 samples). Values refer to the RA of each (sub)species shown in relation to the complete microbiota profile (i.e., all bacterial taxa). M = month.

Table S7. Association of maternal stool *B. infantis* detection (in the first week postpartum) and infant *B. infantis* absolute abundance (AA) during the first month of age. [a]

	Difference in infant *B. infantis* AA Regression coefficient (95% CI)			
**Variable**	**Unadjusted**	**Adjusted for delivery mode**	**Adjusted for study site**	**Adjusted for delivery mode and study site**
Maternal *B. infantis* detection (above LOD)	5.60 (5.11, 6.08)	0.49 (-0.17, 1.16)	5.60 (5.11, 6.08)	0.49 (-0.17, 1.16)
C-section	-	5.28 (4.74, 5.82)	-	5.28 (4.73, 5.83)
Study site (MFSTC)	-	-	0.00 (-0.043, 0.043)	0.00 (-0.095, 0.095)

[a] All coefficients were based on a quantile regression model with clustered standard errors. Maternal stool *B. infantis* was treated as a time-fixed, independent binary (detected vs not-detected) variable based on the qPCR result of the first maternal stool sample available within 0-7 days postpartum. Infant *B. infantis* AA was treated as the dependent, continuous variable. Analysis was restricted to infant samples collected in the first month of age (3-31 days) and included only infant samples whose mothers also had a sample collected. Infant samples collected prior to their mother sample were not included in the analysis (n=1481 samples, 429 infants). LOD: denotes limit of detection.

Table S8. qPCR primers and probes employed in this study.

Target	Name	Sequence (5' to 3') [a]	Length	Reference
B. longum subsp. *infantis*	inf_2348_F (Forward)	ATA CAG CAG AAC CTT GGC CT	20	Lawley et al 2017 (3)
	inf_2348_R (Reverse)	GCG ATC ACA TGG ACG AGA AC	20	
	inf_2348_PB (Probe)	/6-FAM/ TTT CAC GGA /ZEN/ TCA CCG GAC CAT ACG /3IABkFQ/	24	
	PhHV_F (Forward)	GGG CGA ATC ACA GAT TGA ATC	21	
	PhHV_R (Reverse)	GCG GTT CCA AAC GTA CCA A	19	
	PhHV_seq1 (probe)	5'-Cy5 /TAT GTG TCC GCC ACC ATC T/3BHQ	19	
B. longum subsp. *longum*	lon_0274_F (Forward)	GAG GCG ATG GTC TGG AAG TT	20	Lawley et al 2017 (3)
	lon_0274_R (Reverse)	CCA CAT CGC CGA GAA GAT TC	20	
	lon_0274_PB (Probe)[b]	/5TexRd-XN/ AAT TCG ATG CCC AGC GTG GTC TT /BHQ-2/	23	
B. breve	bre_1623_F (Forward)	GTG GTG GCT TGA GAA CTG GAT AG	23	Haarman et al 2002 (4)
	bre_1623_R (Reverse)	CAA AAC GAT CGA AAC AAA CAC TAA A	25	
	bre_1623_PB (Probe)	/6-FAM/ TGA TTC CTC GTT CTT GCT GT /MGB-NFQ/	20	

[a] *B. infantis, B. longum,* and *B. breve* primers were produced by ACGT Inc. (Toronto, ON, Canada); *B. infantis*, *B. longum*, and PhHV probes were produced by Integrated DNA technologies Canada Inc (IDT) (Toronto, ON, Canada); *B. breve* and PhHV primers were produced by Invitrogen Life Technologies™ (Burlington, ON, Canada).

[b] Probe modified from original design: 6-FAM reporter replaced with 5TexRd-XN, 3IABkFQ replaced with BHQ-2 and internal ZEN quencher removed.

Abbreviations: 6FAM = 5' 6-FAM™ reporter; ZEN = Internal ZEN™ quencher; IABkFQ = 3' Iowa Black® FQ quencher; Cy5 = Cy5 dye; 3BHQ = Black Hole quencher 3; 5TexRd-XN = 5' Texas Red-X (NHS Ester) reporter; BHQ-2 = 3' Black Hole Quencher-2; MGB-NFQ = Minor Groove Binder – 3' Nonfluorescent Quencher.

Supplemental references

1. WHO. 2015. (World Health Organization) Guideline: managing possible serious bacterial infection in young infants when referral is not feasible. World Health Organization, Geneva. https://iris.who.int/handle/10665/181426.

2. Filmer D, Pritchett LH. 2001. Estimating wealth effects without expenditure data-or tears: an application to educational enrollments in states of India. Demography 38:115–132.

3. Lawley B, Munro K, Hughes A, Hodgkinson AJ, Prosser CG, Lowry D, Zhou SJ, Makrides M, Gibson RA, Lay C, Chew C, Lee PS, Wong KH, Tannock GW. 2017. Differentiation of *Bifidobacterium longum* subspecies *longum* and *infantis* by quantitative PCR using functional gene targets. PeerJ 5:e3375.

4. Haarman M, Knol J. 2005. Quantitative real-time PCR assays to identify and quantify fecal *Bifidobacterium* species in infants receiving a prebiotic infant formula. Appl Environ Microbiol 71:2318–2324.
