# Supplementary figures and images for: Abundance of Bifidobacterium species in the infant gut microbiota and associations with maternal-infant characteristics in Dhaka, Bangladesh
Source: mSphere. 2025 Sep 15;10(10):e00314-25. doi: 10.1128/msphere.00314-25 (PMC12570478; doi:10.1128/msphere.00314-25)

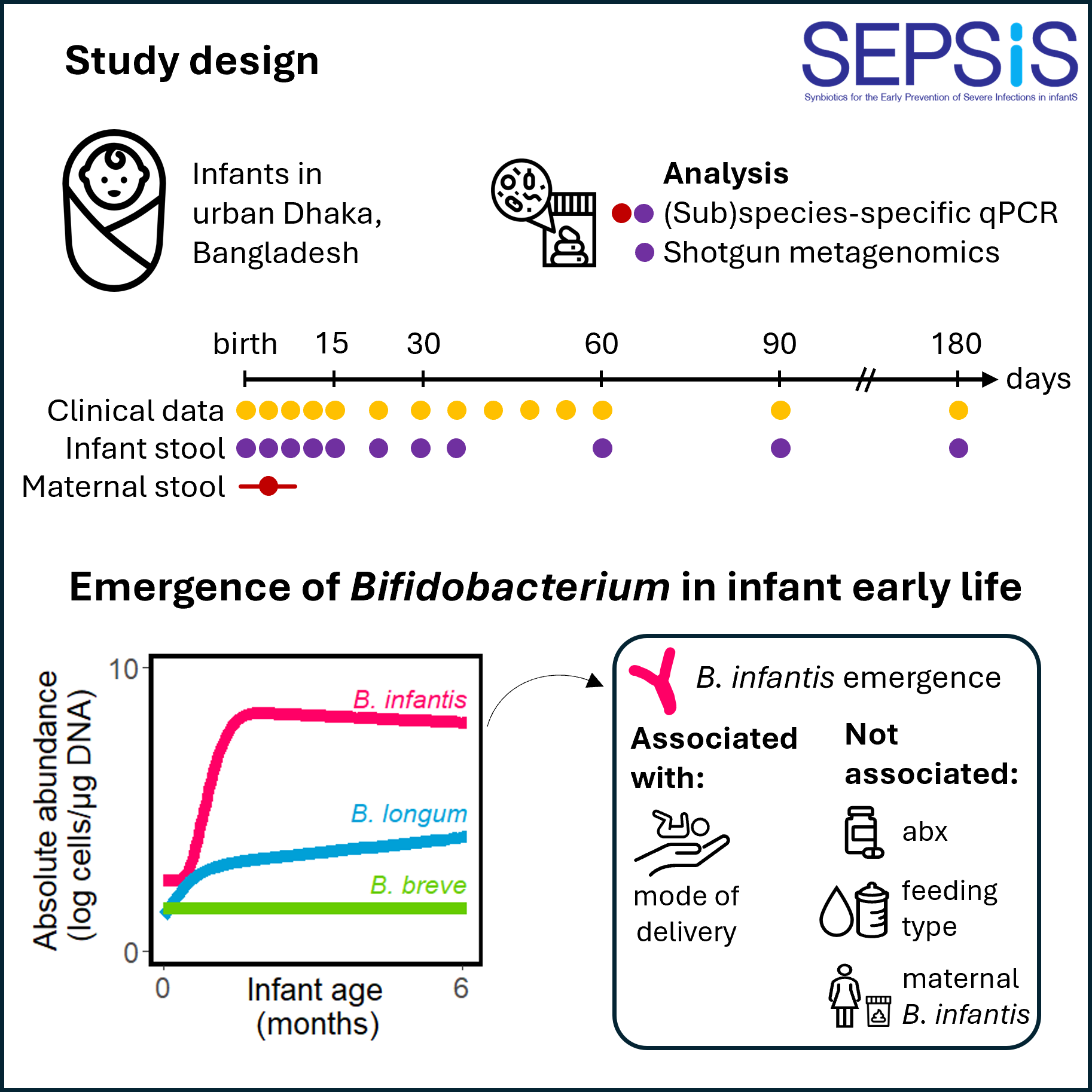

Supplement: Abstract — Graphical abstract. [file msphere.00314-25-s0002.tif]
